# Differential Wide Temperature Range CMOS Interface Circuit for Capacitive MEMS Pressure Sensors

**DOI:** 10.3390/s150204253

**Published:** 2015-02-12

**Authors:** Yucai Wang, Vamsy P. Chodavarapu

**Affiliations:** Department of Electrical and Computer Engineering, McGill University, McConnell Engineering Building, 3480 University Street, Montreal, QC H3A 0E9, Canada; E-Mail: yucai.wang@mail.mcgill.ca

**Keywords:** CMOS sensor circuits, capacitance measurement, MEMS pressure sensor, wide temperature electronics, high temperature sensors, capacitance to voltage converter

## Abstract

We describe a Complementary Metal-Oxide Semiconductor (CMOS) differential interface circuit for capacitive Micro-Electro-Mechanical Systems (MEMS) pressure sensors that is functional over a wide temperature range between −55 °C and 225 °C. The circuit is implemented using IBM 0.13 μm CMOS technology with 2.5 V power supply. A constant-*g_m_* biasing technique is used to mitigate performance degradation at high temperatures. The circuit offers the flexibility to interface with MEMS sensors with a wide range of the steady-state capacitance values from 0.5 pF to 10 pF. Simulation results show that the circuitry has excellent linearity and stability over the wide temperature range. Experimental results confirm that the temperature effects on the circuitry are small, with an overall linearity error around 2%.

## 1. Introduction

Micro-Electro-Mechanical Systems (MEMS) pressure sensors are widely used in various applications including automobiles, industrial process control, avionics and aerospace, consumer electronics, and oil and gas extraction [1,2]. In recent years, MEMS pressure sensors have moved from piezoresistive bridges to hermetically sealed capacitive devices that offer advantages of high sensitivity, wide dynamic range, and low power consumption [3,4]. MEMS capacitive pressure sensors typically have a steady-state capacitance on the order of few picoFarads and during operation provide a capacitance change on the order of few tens of femtoFarads. There are many commercially available capacitance to digital converters that interface with MEMS sensors from Analog Devices Inc. (Model: AD7152) [5] and ISC8 Inc. (Model: MS3110) [6] that work over the commercial temperature range from −40 °C to 85 °C.

In many industrial applications, MEMS pressure sensors operate over a wide temperature range from −55 °C to 225 °C [7]. Further, there are applications at higher temperatures that reach 400 °C [8], but those are beyond the reach of sensing circuits made using Complementary Metal-Oxide Semiconductor (CMOS) process. Development of sensor interface electronics at high temperatures is challenging as high temperatures cause several detrimental effects in CMOS integrated circuits including threshold voltage drop, carrier mobility reduction and increase of junction leakage current which could cause chip failure due to latch-up. Given the minute capacitance output from capacitive MEMS pressure sensors that are on the order of few femtoFarads to few picoFards, the interface electronics must ideally be located in close proximity to the sensor devices to maintain good signal to noise ratio and reduce wiring complexity. Looking at the wide temperature range from −55 °C to 225 °C, there are various available design techniques to mitigate these high temperature effects in CMOS electronics [9]. Using these techniques, many research groups have implemented high temperature interface circuits for piezoresistive and piezoelectric MEMS sensors [10–12]. However, there is no available wide temperature range CMOS sensor interface circuit for capacitive MEMS sensors implemented to date. Hence, new sensor interface electronics are required to be developed that are capable of operating over a wide temperature range that minimize the need for extensive and cumbersome heat sinks while providing high sensitive capacitance measurement.

In this paper, we present a novel CMOS differential interface circuit for capacitive MEMS pressure sensors. This capacitance-to-voltage converter circuit is designed to operate over a temperature range between −55 °C and 225 °C. It is implemented in IBM 0.13 μm CMOS technology with 2.5 V power supply. The circuit offers the flexibility to interface with MEMS sensors with a wide range of the steady-state capacitance values from 0.5 pF to 10 pF. In Section 2, we describe the design of the CMOS sensor interface circuit with constant-*g_m_* biasing. In Section 3, we describe the experimental measurements and discussion. Finally, we provide conclusions for the research work.

## 2. Design of CMOS Sensor Interface Circuit

**Figure 1.**
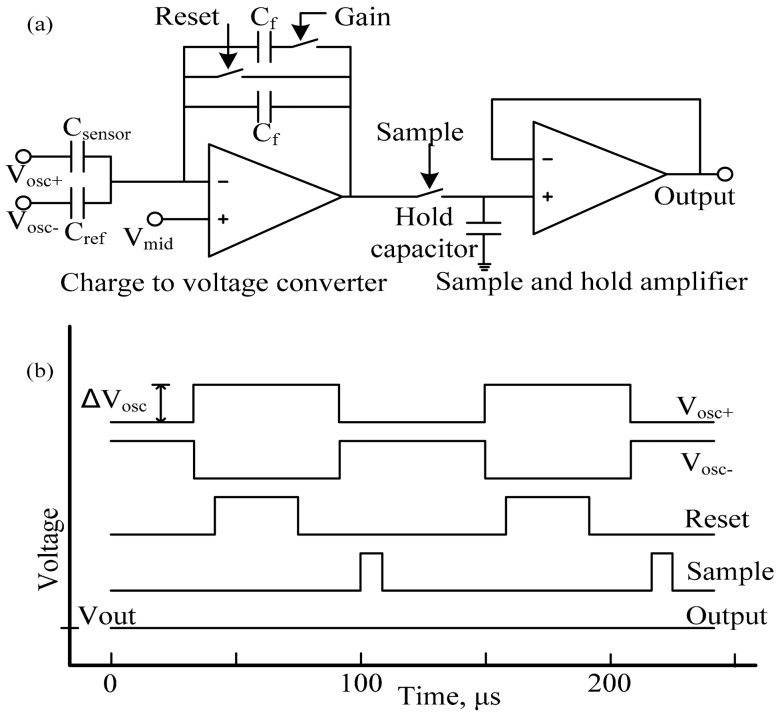
(**a**) Schematic diagram of the Complementary Metal-Oxide Semiconductor (CMOS) sensor interface circuit; (**b**) Waveforms of the control and output signals for the CMOS circuit.

Figure 1a illustrates the schematic diagram of the differential wide temperature CMOS interface circuit for capacitive MEMS pressure sensors. The circuit uses a switched capacitor technique and a similar circuit has been used previously by our group [13] for MEMS air flow sensors and by Patel *et al.* [14] towards chemocapacitive sensing of volatile organic compounds. Here, we use a novel differential input circuit that enables working with MEMS sensors that have a wide range of the steady-state capacitance values from 0.5 pF to 10 pF. The circuit measures the capacitance of external sensor, C_sensor_, and produces an analog output voltage, Vout. A reference capacitor, C_ref_ is needed that has a similar steady-state capacitance value as the sensor device, C_sensor_, to extend the detection dynamic range while providing good capacitance detection resolution required for MEMS pressure sensors.

In operation, two 10 kHz square wave signals V_OSC+_ and V_OSC−_ with same amplitude but opposite phase are used to drive the sensor capacitor, C_sensor_ and reference capacitor, C_ref_. Their amplitude is defined as ΔV_osc_. V_mid_ is a DC voltage applied to the non-inverting node of the amplifier. Reset, Sample and Gain are control signals where the Gain switch can adjust the feedback capacitance in the circuit, C_fedback_, to be either C_f_ or 2C_f_. In the present design, the values of C_f_ and the hold capacitor, C_H_ are both set as 1 pF. The waveforms of the control and output signals for the converter circuit are shown in Figure 1b. The amplitude of the output voltage can be expressed as Equation (1),

(1) $${\text{V}}_{\text{out}}={\text{V}}_{\text{mid}}+\Delta {\text{V}}_{\text{osc}}\frac{\left({\text{C}}_{\text{sensor}}-{\text{C}}_{\text{ref}}\right)}{{\text{C}}_{\text{feedback}}}$$

**Figure 2.**
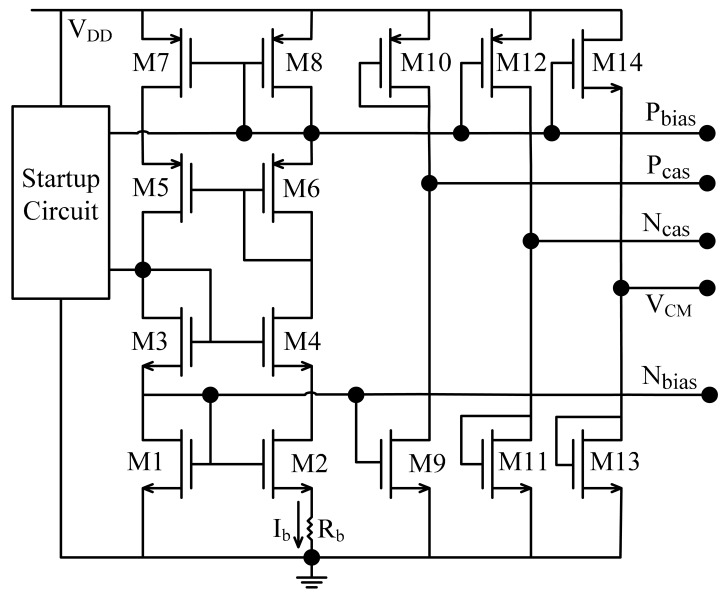
Schematic of the constant-*g_m_* biasing circuit.

At high temperatures, the overall performance of operational amplifiers implemented in CMOS process degrade greatly, most notably by showing loss of open loop gain and decrease of bandwidth. Effective design techniques are required to compensate high temperature effects and reduce the impact of excessive leakage current [9,15,16] Amongst different high temperature compensation techniques, the constant-*g_m_* biasing technique is a popular choice which has been proven effective over the wide temperature range [17,18]. A biasing circuit using constant-*g_m_* biasing technique is shown in Figure 2. When neglecting channel length modulation and the body effect, the *g_m_* of a transistor biased by current, I_b_ as shown in Figure 2 can be expressed as Equation (2),

(2) $$g_{m}=\frac{2}{{\text{R}}_{\text{b}}}\sqrt{\frac{\text{W}}{\text{L}}}\left(\sqrt{\frac{{\text{L}}_{1}}{{\text{W}}_{1}}}-\sqrt{\frac{{\text{L}}_{2}}{{\text{W}}_{2}}}\right)$$

As can be seen, the *g_m_* of the transistor is inversely proportional to the biasing resistor, R_b_. Ideally, if R_b_ has a zero Temperature Coefficient (TC), then *g_m_* will be constant as the temperature changes. The IBM 0.13 μm CMOS technology offers a resistor with TC around 100 ppm/°C. The biasing circuit when implemented with this resistor can achieve considerably high stability over a wide temperature range. A folded-cascode amplifier with a switched-capacitor common-mode feedback (CMFB) circuit is used as the amplifier in Figure 1a [13]. A constant-*g_m_* biasing circuit is used to bias the amplifier to stabilize the amplifier performance (open loop gain and bandwidth) over the wide temperature range. As the open loop gain decreases when temperature increases, a higher gain amplifier is necessary, such that it still maintains sufficient gain at high temperatures. A NMOS input pair is used in our design to attain large gain while keeping the device sizes reasonably small.

For the chip layout, common-centroid structures are used wherever applicable to reduce temperature induced common mode errors. Guard rings are placed around performance sensitive blocks to prevent latch-up at high temperatures.

## 3. Experimental Results and Discussion

**Figure 3.**
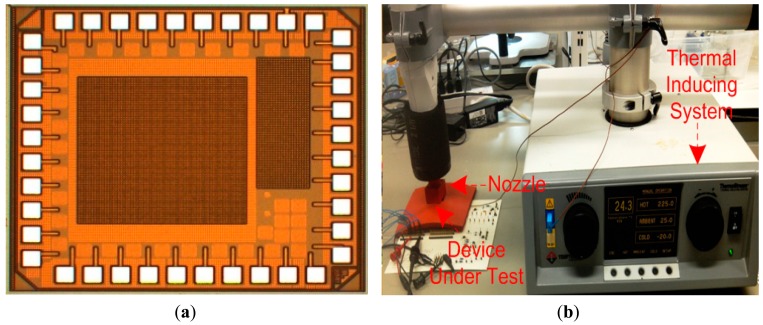
(**a**) Chip microphotograph measuring 1950 mm × 1850 mm, where the effective area of the CMOS sensor interface circuit is 460 μm × 290 μm; (**b**) Experimental setup of Thermostream TP04100A thermal inducing system.

**Figure 4.**
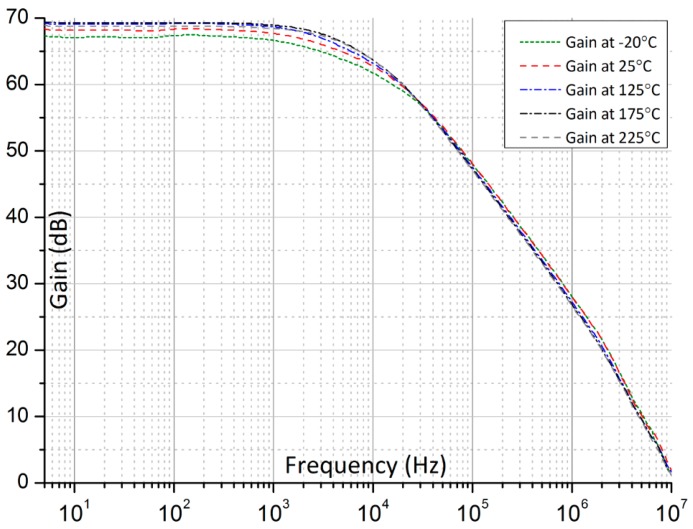
Measured open-loop frequency response of the folded-cascode amplifier at different temperatures.

The CMOS sensor interface circuit is fabricated using the IBM 0.13 μm CMOS process available through the Canada Microelectronics Corporation. The microphotograph of the fabricated chip is shown in the inset of Figure 3a. The chip is placed under the nozzle of a thermal inducing system (Thermostream TP04100A, Temptronic Corp., Mansfield, MA, USA), where the chip temperature can be varied from −20 °C to 225 °C, which is the temperature limit of the Thermostream testing system as shown in Figure 3b. The temperature under the nozzle is monitored using a thermocouple and the temperature accuracy is better than ±1 °C. Figure 4 shows the measured single-ended open-loop gain of the amplifier driving 2 pF load capacitance at different temperatures. From −20 °C to 225 °C, the measured DC gain varies by only 2.1 dB. The measured amplifier power consumption (including constant-*g_m_* biasing circuit) at 225 °C is 0.51 mW. We tested three out of five received packed chips and all showed similar frequency response *versus* temperatures.

**Figure 5.**
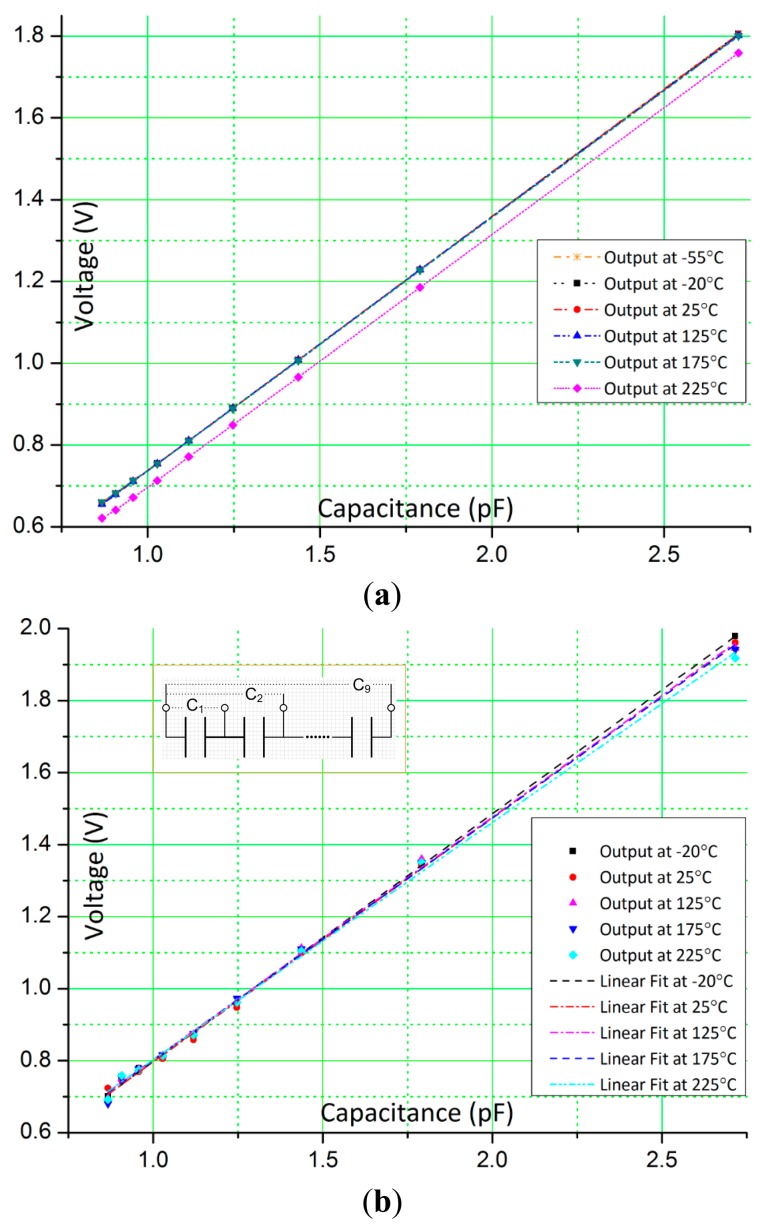
Output voltages of the converter circuit at different temperatures. (**a**) Simulated output voltage; (**b**) Measured output voltage. Inset: schematic of the test C_sensor_.

Figure 5a,b show the output voltages for different capacitance values during calibration at temperature ranges, simulated from −55 °C to 225 °C and measured from −20 °C to 225 °C. Again, the measured temperature range is less due to the limit of the Thermostream testing system. Generally, colder temperatures between −55 °C and −20 °C do not have detrimental effects on CMOS electronics and further help to improve signal to noise ratio due to reduced noise effects. The calibration capacitance, C_sensor_, is constructed by using nine capacitors, C_1_ to C_9_ (shown in inset of Figure 5b) with nominal capacitance value of 1 pF which are connected in series. By appropriately changing connections from C_1_ to C_9_, the capacitance value of C_sensor_ is varied and measured using a high resolution universal capacitance readout circuit, MS3110 (MicroSensors, Costa Mesa, CA, USA) that is maintained at room temperature (25 °C).

**Figure 6.**
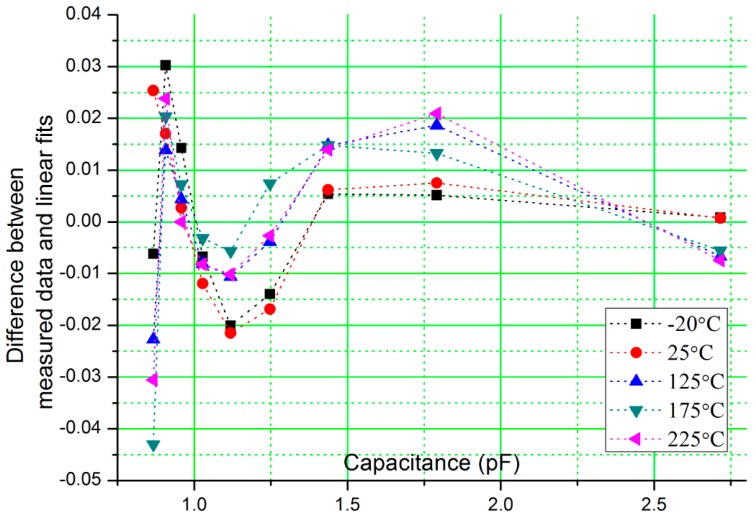
Difference between measured results and linear fits.

The control signals, V_osc+_, V_osc−_, Reset and Sample are generated by a data acquisition card, NI USB-6259 (National Instruments Inc., Austin, TX, USA). The amplitude of V_osc_ is set to 1.25 V, V_mid_ is set to analog ground 1.25 V, the feedback capacitance, C_f_ is set to 2 pF, and the reference capacitance, C_ref_ is fixed to 1.805 pF. The sensor capacitor, C_sensor_, and reference capacitor, C_ref_, are placed at room temperature (25 °C) with only the test chip which includes the on-chip feedback capacitor C_f_ exposed to the temperature change from −20 °C to 225 °C. From Figure 5, the measured results show similar response as simulated results. Over the wide temperature range, the circuit converts the input capacitance to output voltage with goodness-of-fit of linear regression of 0.998 (*R*^2^). Figure 6 shows the difference between the measured results and the linear fits, which is defined as, (measured value-fitted value)/(fitted value). The overall difference is around 2%, and larger difference is observed when the sensor capacitance is smaller and at high temperatures. The tested linearity is worse than the simulated results, especially when the capacitance is smaller and its probable cause could be interconnect parasitic capacitance in the experimental setup.

**Figure 7.**
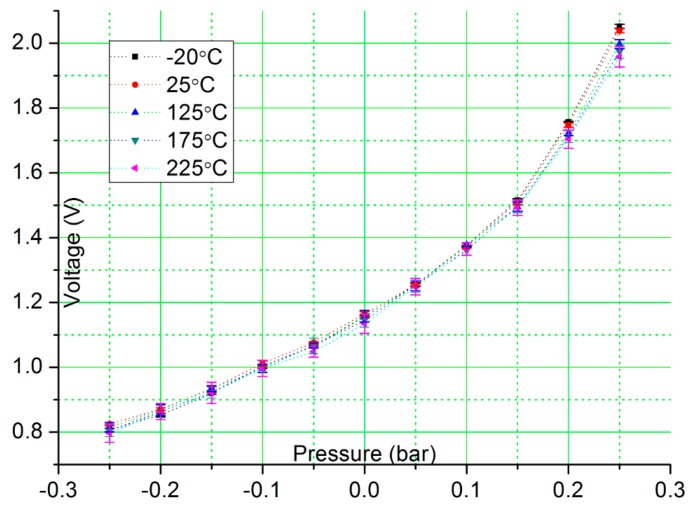
Measured output response of the CMOS interface circuit with a capacitive MEMS pressure sensor.

The CMOS circuit is interfaced with a capacitive MEMS pressure sensor from Protron Mikrotechnik GmbH, Germany [19] by replacing the C_sensor_ with the pressure sensor in Figure 1. The feedback capacitance, C_f_ is set to 2 pF. The sensor operates within a temperature range from −40 °C to +85 °C with a sensitivity of 1 fF/mbar. During our experimental testing, the MEMS pressure sensor is maintained at room temperature (25 °C) and placed in a pressure vessel so that its capacitance output does not change with ambient temperature and only varies when external pressure is applied to the pressure vessel. Figure 7 shows the measured output voltage to pressure change when the CMOS sensor interface circuit is exposed to temperature range from −20 °C to 225 °C. The MEMS pressure sensor is exposed to pressure variation from −0.25 bar to +0.25 bar. All measurements are done in triplicate with the average value noted in Figure 7. As can be seen, the output voltage from the CMOS circuit changes in accordance with the capacitance change of the pressure sensor and provides a stable response over a temperature range from −20 °C to 225 °C. The estimated sensor readout resolution is 2.322 mV/mbar at 225 °C. As temperature increases, the output noise becomes larger. The simulated noise analysis shows the noise level at the output of the circuit in Figure 1a increases from 0.134 mVHz^−1/2^ to 0.184 mVHz^−1/2^ when temperatures changes from −55 °C to 125 °C. Another major error source at high temperatures is the leakage current in the Sample switch, which can cause a voltage drop or rise on the hold capacitor, depending on the direction of the leakage current. This problem can be alleviated using higher sampling speed, or increasing the capacitance of the hold capacitor. A sample/hold circuit with dynamic switch leakage compensation [20] can also be used, with the penalty of increased circuit complexity.

In our experiments, the temperature for the sensor and reference capacitors is fixed at 25 °C. In real world applications, the sensor capacitor and reference capacitors will also undergo wide temperature change. The sensor capacitor and reference capacitor are often identical and they usually have the same TCs, around 100 ppm/°C. The TC of the on-chip feedback capacitor is about 13 ppm/°C according to the IBM 0.13 μm CMOS process design manual. According to Equation (1), some errors could be introduced due to the TC difference. The sensor capacitance, C_sensor_ is comprised of a steady-state capacitance defined as C_0_ and a capacitance change during operation defined as, ΔC. And the reference capacitance, C_ref_ is usually about the same value of C_0_. Now Equation (1) can be written as:

(3) $${\text{V}}_{\text{out}}={\text{V}}_{\text{mid}}+\Delta {\text{V}}_{\text{osc}}\frac{\Delta \text{C}}{{\text{C}}_{\text{feedback}}}$$

**Figure 8.**
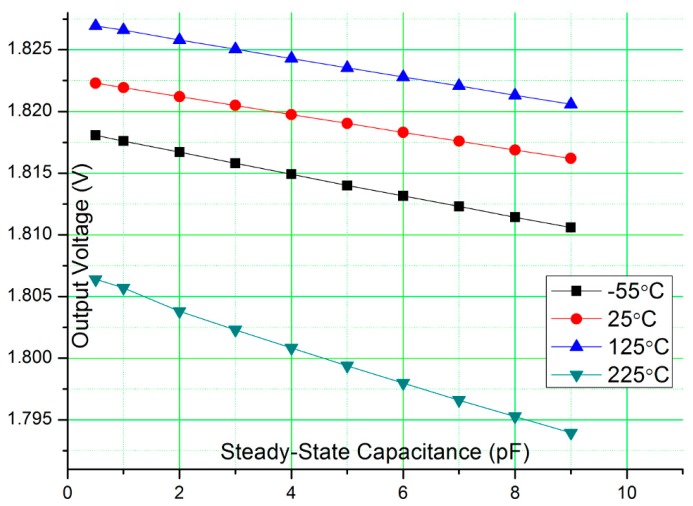
Simulated output voltage for different steady-state capacitance.

As can be seen, due to the differential readout scheme, the C_0_ component is cancelled and only ΔC is considered. Figure 8 shows the simulated output voltage for different steady-state capacitance from −55 °C to 225 °C. The TCs of sensor capacitor and reference capacitor are set to 100 ppm/°C. ΔC is set to 0.5 pF at 25 °C, V_mid_ and ΔV_osc_ are set to 1.25 V, and C_feedback_ is set to 1 pF. As can be seen, when the steady-state capacitance changes from 0.5 pF to 10 pF, the output voltages decrease only about 20 mV. This shows the circuit has good flexibility to interface with MEMS sensors with a wide range of the steady-state capacitance values. At a fixed steady-state capacitance, the output voltages change by less than 30 mV when temperature varies from −55 °C to 225 °C. This means the TC difference has very limited impact to the interface circuit. For MEMS sensors which do not have a reference capacitor, low TC mica capacitor (with TC about 50 ppm/°C) can be used as reference capacitor.

## 4. Conclusions

A CMOS differential interface circuit for capacitive MEMS pressure sensors is designed, fabricated and tested that is functional over a wide temperature range between −55 °C and 225 °C. The circuit is implemented using IBM 0.13 μm CMOS technology with 2.5 V power supply. Experimental results show that the circuitry has excellent temperature stability and an overall linearity error of around 2% over the wide temperature range with goodness-of-fit of linear regression of 0.998 (*R*^2^). The circuit was tested using a commercial capacitive MEMS pressure sensor and provided a stable response over the wide temperature range.
